# Cardiopulmonary exercise testing and elective open abdominal aortic aneurysm surgery over a 6-year period in a UK teaching hospital

**DOI:** 10.1186/cc11074

**Published:** 2012-03-20

**Authors:** AH Raithatha, S Smith, K Chakrabarti, A Tridente, K Kerr

**Affiliations:** 1 Sheffield Teaching Hospitals NHS Trust: Northern General Hospital, Sheffield, UK

## Introduction

A reduced oxygen uptake at anaerobic threshold (AT) and an elevated ventilatory equivalent for carbon dioxide (VE/VCO_2_) have been shown to be predictors of outcome after major surgery [1]. We report the demographic and outcome data of patients undergoing elective open abdominal aortic aneurysm (AAA) surgery who underwent cardiopulmonary exercise testing (CPET) testing within our unit and examine the relationship between age, AT and VE/VCO_2 _on survival outcomes.

## Methods

A retrospective observational analysis of our unit's CPET Excel database was conducted to identify patients who underwent CPET testing for elective open AAA repair over a 6-year period. Demographic data and survival at 30 days, 90 days and 1 year were extracted. Logistic regression analysis was undertaken using STATA statistical software to determine if age, AT or VE/VCO_2 _were predictors of survival at 30 days, 90 days or 1 year.

## Results

**Figure 1:**
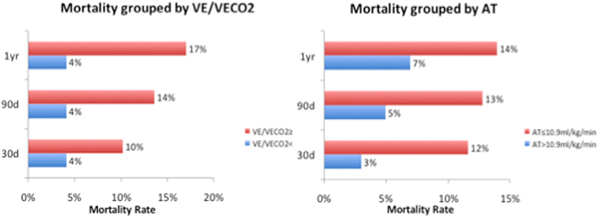
**Elective AAA mortality rates by group**.

CPET was performed in 259 patients who subsequently underwent an elective open AAA repair. Outcome data were available for 185 patients from a potential 222 in whom 1-year follow-up was available (83%). Baseline demographics included AT ≤10.9 ml/kg/ minute in 39% and >10.9 ml/kg/minute in 61% of patients with respective median ages in these groups being 73 and 72. Regression analysis demonstrated that AT was the only predictor of survival at 30 days, 90 days and 1 year. Age and AT remained independent predictors of survival at 90 days and 1 year following multivariate analysis. Of note, 87 patients underwent elective endovascular aneurysm repair and CPET, median age 76, during the period analysed. In particular, 26.4% were older than 80 years old, versus 14.7% in the AAA group. See Figure 1.

## Conclusion

Our data support existing evidence that AT can be used as a predictor of survival in open elective AAA surgery. In addition, age at CPET also predicted 90-day and 1-year survival; however, VE/VCO_2 _was not a predictor of survival in this cohort.
